# MPO-ANCA-associated necrotizing glomerulonephritis in rheumatoid arthritis; a case report and review of literature

**DOI:** 10.15171/jnp.2017.10

**Published:** 2016-10-27

**Authors:** Mário Góis, Ana Messias, Dulce Carvalho, Fernanda Carvalho, Helena Sousa, João Sousa, Fernando Nolasco

**Affiliations:** Nephrology Department, Hospital Curry Cabral, Centro Hospitalar de Lisboa Central, Lisboa, Portugal

**Keywords:** ANCA, Vasculitis, Rheumatoid arthritis, Rituximab, Necrotizing glomerulonephritis

## Abstract

**Background:**

Renal involvement in rheumatoid arthritis (RA) is common and has a negative impact on patient survival. Only few cases have been reported of necrotizing glomerulonephritis (GN) associated with myeloperoxidase anti-neutrophil cytoplasmic antibody (MPO-ANCA) in patients with RA.

**Case Presentation:**

We report a patient with RA who developed a necrotizing GN associated with ANCA-MPO, treated with rituximab (RTX). A 55-year-old man with a 27-year history of RA under secukinumab was referred to our nephrology clinic with worsening renal function associated with microhematuria and proteinuria. Our laboratory evaluation showed hypocomplementemia and positive titers for MPO-ANCA (615 U/mL). A renal biopsy demonstrated pauci-immune necrotizing GN. The patient was treated with 3 consecutive pulses of methylprednisolone followed by oral prednisolone (1 mg/Kg) and rituximab (1000 mg, repeated 14 days later). After a 10-month follow-up, the arthritis remains well-controlled, renal function stabilized, proteinuria improved and MPO-ANCA titer normalized (6.3 U/mL).

**Conclusions:**

Necrotizing GN is a rare but a serious condition and an early diagnosis is essential to treatment. This is the first case of necrotizing GN (without extra-renal manifestations of vasculitis) in a patient with active RA, successfully treated with RTX.

**Implication for health policy/practice/research/medical education:**

RA is a systemic inflammatory disorder and renal involvement has a negative impact on prognosis. We describe the first reported patient with RA, presenting with necrotizing GN and without extra-renal signs of vasculitis, successfully treated with RTX.

## 1. Introduction

Rheumatoid arthritis (RA) is a chronic systemic inflammatory disease that primarily affects joints. Renal involvement of RA has a wide spectrum of lesions, consisting of glomerular damage due, mainly, to secondary amyloidosis and membranous nephropathy (related to gold salts, D-penicillamine) and also tubular damage (due to analgesic and nonsteroidal anti-inflammatory drugs [NSAIDs]). Necrotizing glomerulonephritis (GN) associated with myeloperoxidase anti-neutrophil cytoplasmic antibody (MPO-ANCA) is a rare complication (1). We report a patient with RA and pauci-immune necrotizing GN associated with MPO-ANCA.

## 2. Case Report

**Figure 1:**
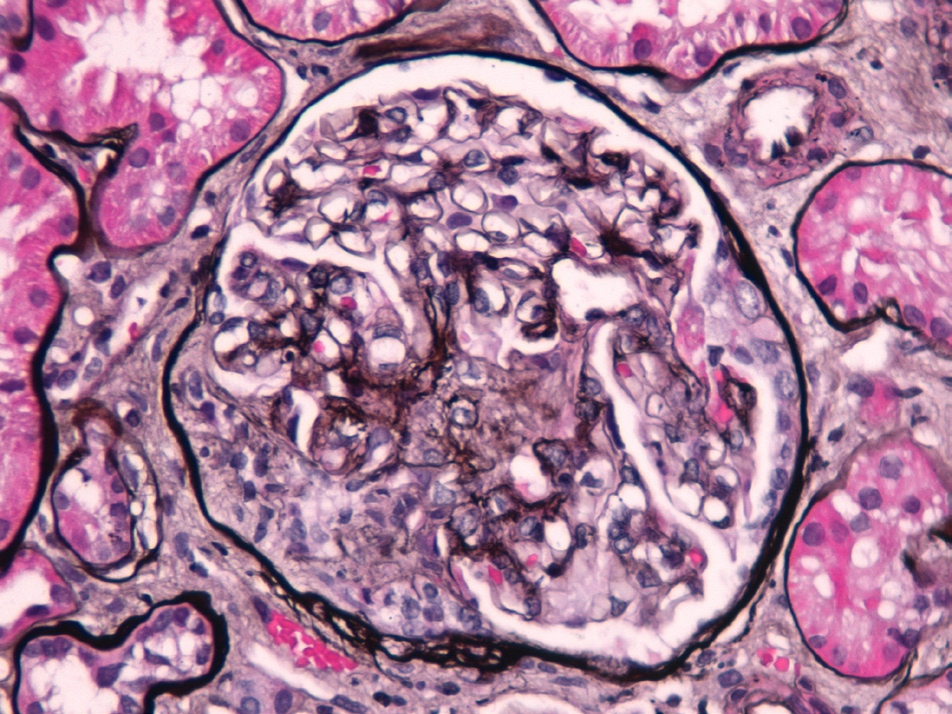
Renal biopsy, methenamine silver; X400. The kidney biopsy specimen contained 13 glomeruli and one had segmental necrosis and cellular crescent formation.

The patient was a 55-year-old male with a history of seropositive RA since 1988. The patient was previously treated with disease-modifying anti-rheumatic drugs (DMARDs), including cyclosporine, methotrexate and monoclonal antibodies against tumour necrosis factor-alpha (anti-TNF-α) – adalimumab, followed by infliximab – with poor disease control. After one year of treatment with an anti-interleukin 17A (IL-17A) monoclonal antibody (secukinumab), being in a clinical trial, the patient was referred to our nephrology clinic with worsening renal function (serum creatinine [sCr] rising from 0.7 to 3.1 mg/dL) associated with microhematuria and proteinuria (2.6 g/24 h). Analysing the blood and urine results from the year before, microhematuria and proteinuria had been present since the beginning of secukinumab, and serum creatinine started rising in the 6 months before referral. At this time, the patient still had active arthritis of the elbows and wrists. Our evaluation showed hemoglobin 10.2 g/dL, erythrocyte sedimentation rate 62 mm/h, hypocomplementemia (C3 0.67 g/L and C4 0.09 g/L, normal range 0.9–1.8 and 0.1–0.4, respectively) and positive titers for MPO-ANCA (615 U/mL, normal range < 20) and anti-citrullinated peptide antibodies (CCP) (> 300 U/mL, normal range < 12). Chest X-ray was normal. A renal biopsy was performed. The kidney specimen contained 13 glomeruli, 5 of these exhibited crescents (one with fibrinoid necrosis), including 2 cellular crescents (Figure 1) and 3 fibrocellular crescents. The biopsy also showed fibrosis occupying 30% of the interstitium with minimal lymphoplasmocytic infiltrate. All vessels were normal with robust internal elastic lamina. By immunofluorescence there were no deposits of IgA, IgM, IgG, C3, C1q or fibrin. Thus, the diagnosis of pauci-immune necrotizing GN was made. The patient was treated with three consecutive pulses of methylprednisolone followed by oral prednisolone (1 mg/kg) and rituximab (RTX) according to RA’s scheme (1000 mg repeated 14 days later). Two months later, the patient had an important clinical improvement with full remission of joint pain, as well as an improvement in renal function (sCr 1.68 mg/dL), proteinuria (1.6 g/24 h) and a reduction in MPO-ANCA titer (51.10 U/mL) and normalization of complement levels (C3 1.2 g/L and C4 0.26 g/L). At the last evaluation, 10 months later, the prednisolone dose was tapered at 5 mg/d and arthritis remains well-controlled, renal function stabilized (sCr 1.73 mg/dL), proteinuria improved (500 mg/24 h) and MPO-ANCA titer normalized (6.3 U/mL).

## 3. Discussion

We described a patient with RA who developed a pauci-immune necrotizing GN. The renal involvement in patients with RA is complex and includes the lesions caused by therapy (DMARDs and NSAIDs). Moreover, renal lesions may be a consequence of the duration and severity of the disease, such as secondary amyloidosis (1). Necrotizing GN is a rare complication of RA. It may occur as a manifestation of RA-associated vasculitis (with a multi-organic involvement, affecting the eye, nose, throat, lung and skin) or it may appear as an isolated disorder (2). Makino et al reported the results of renal biopsies in 100 patients with RA. These patients had a great heterogeneity of renal lesions, and the most frequent finding was membranous nephropathy (n = 31) and only 6 of these cases were considered not related to DMARDs (3). Renal amyloidosis was detected in 11 patients and only 2 patients had crescentic GN. Previously, Nakano et al reported a frequency of 0.6% of necrotizing GN, in an analysis of renal biopsies of 158 patients with RA (4) and, in literature, there are few published cases of renal vasculitis in these patients. Our patient presented with exclusive renal manifestations and without evidence of extraglomerular vasculitis on kidney biopsy.

The development of the overlap syndrome of RA and vasculitis may be explained on basis of the common pathogenic pathways. Mustila et al published a series of 246 patients with RA and ANCAs were found in 21% (n = 52). This study also showed that ANCAs were associated with disease activity and severity and were an independent predictor of RA-associated nephropathy (5). The classical target antigens of ANCAs are: proteinase-3 (PR3), highly associated with granulomatosis with polyangiitis (6), and MPO, mainly related with microscopic polyangiitis(7). In patients with RA the most common is MPO-ANCA. However, other antigens have been detected in sera from patients with RA, such as elastase, lactoferrin and cathepsin G (8), remaining the controversy about their significance and specificities in RA. In our patient high titers of MPO-ANCAs were found and related to disease activity. The pathogenic role of ANCAs in this overlap syndrome is suggested, but it will be more beyond the ANCAs, like a genetic predisposition to autoimmunity, which involves genes such as *PTPN22* (9) and polymorphisms in uteroglobin and NF-κB2(10).

Yoshihara et al described three patients with RA with rapidly progressive renal failure and high titers of MPO-ANCA. One patient had crescentic GN proved by renal biopsy. Three patients did not respond to therapy, including steroids, intensive immunosuppressive therapy and plasma exchange, and underwent hemodyalisis(11).

Harper et al reported 10 patients with RA who developed focal segmental necrotizing GN. At presentation, all patients had renal dysfunction and four patients were positive for MPO-ANCA. Six patients were treated with prednisolone and cyclophosphamide, two patients with prednisolone and azathioprine and two patients only with prednisolone. At a 5-year follow-up, four patients died, one remains dialysis-dependent and four have stable renal function (median creatinine 148.5 μmol/L)(12).

Szilasi et al described four patients with RA and ANCA-associated vasculitis (three positive for MPO-ANCA and one positive for PR3-ANCA). Two patients had renal involvement proved by biopsy and histology showed a crescentic GN. Of these, one patient underwent hemodyalisis, in spite of all the immunosuppressive therapy administered (steroids, cyclophosphamide) and also plasma exchange. The other patient remains with stable renal function (glomerular filtration rate of 54 mL/min) and PR3-ANCA is negative (1.7 U/mL)(13).

Draibe and Salama reported six patients who had a diagnosis of RA and developed ANCA-associated vasculitis. All the patients had severe renal involvement, with necrotizing GN. Only one patient had the diagnosis of granulomatosis with polyangiitis, with lung infiltrates, maxillary and frontal sinusopathy and positive PR3-ANCA). The other five patients had a clinical diagnosis of microscopic polyangiitis (three were positive MPO-ANCA)(14).

Reitblat and Reitblat reported two patients with ANCA associated vasculitis who was under anti-TNF-α(15). In these cases, it is a challenge to know whether the vasculitis is a complication of the RA or a consequence of the treatment. Our patient only had positive ANCA titers, but is well recognized the immune dysregulation caused by anti-TNF-α, which is associated with the development of autoantibodies, not only ANCA (16), but also antinuclear (ANA), antiphospholipids (17) and anti-double-stranded DNA antibodies (anti-dsDNA) (18). Secukinumab is a fully human IgG1κ anti–IL-17A monoclonal antibody that prevents the binding of IL-17A to its receptor and inhibits the inflammatory response involved in many autoimmune diseases, such as RA, psoriasis and ankylosing spondylitis (19). This cytokine stimulates the synovial fibroblasts and induces the expression of IL-6, IL-8 and matrix metalloproteinases promoting an inflammatory response and cartilage destruction (20). IL-17A in synovial fluid of patients with RA promotes the expressional of RANKL, which plays an essential role in bone reabsorption (21). Secukinumab was recently approved by US Food and Drug Administration (FDA) for psoriasis and ankylosing spondylitis and showed promising results in phase II randomized controlled trials in RA (22,23). In our case, the patient had been treated with secukinumab in the year before. There has been no reported case of vasculitis in patients treated with this drug, but probably secukinumab is not an etiologic agent in this case, as urinary alterations have been present since the beginning of the treatment.

Our patient had a severe renal impairment. Renal function rapidly declined, with microhematuria and proteinuria. One interesting finding in this case, is the presence of hypocomplementemia, with low levels of C3 and slightly low levels of C4. Although it is not a common finding in ANCA-associated vasculitis, it represents a sign of poor prognosis, with higher rates of occurrence of diffuse alveolar hemorrhage, thrombotic microangiopathy and skin lesions(24). Another relevant aspect in this case is the absence of amyloid deposits in kidney, even after, approximately, 30 years since the diagnosis of RA. The incidence of renal amyloidosis in patients with RA varies from 11% to 30%(1,3) and is associated with poor renal outcome(3).

The patient was treated with RTX, a chimeric anti-human CD20, which may be as effective as cyclophosphamide in ANCA associated vasculitis(25). There is evidence that B lymphocytes play a key role in this disease; 1) the proportion of activated B cells is associated with disease activity and severity (26); 2) ANCA antibodies are produced by B cells (27); 3) B cells are the main target of cyclophosphamide, a drug used for many years in this disease and with good results (28). The binding of RTX to CD20 causes depletion of premature and mature B cells and also provokes the inhibition of the interaction between self-reactive B and T cells and increases the regulatory T lymphocyte population (29). In our case, patient received two doses of RTX (1000 mg each) according to RA’s protocol and he achieved disease remission. As maintenance therapy, RTX can be administered according with two protocols: 1) administration of 1000mg every 4-6 months (30); 2) administration according to CD19+ cells repopulation and ANCA titers (31). It is still to prove which is more effective in maintaining the remission, but it is suggested that the possibility of RTX-treated patients having a relapse is markedly low as long as ANCA titers are negative and B-lymphocytes are effectively depleted (32). In our case, after 10 months, the patient was not retreated with RTX for two reasons; there was a good response to RTX – arthritis is well controlled, MPO-ANCA titer is negative, serum creatinine decreased – and CD19+ cells were effectively depleted (0.2 cells/μ). This is the first case reported in literature of a patient with RA and necrotizing GN, without systemic manifestations of vasculitis, which was successfully treated with RTX, in spite of various markers of poor prognosis, such as hypocomplementemia and high MPO-ANCA titers.

## 4. Conclusions

In summary, our case report and our review, reveal that necrotizing GN related to RA is a rare, but a serious condition. Thus, a patient with RA who develops renal impairment and abnormal urinary sediment should promptly be referenced to a nephrologist. The screening of ANCAs should help to detect earlier this disease. The role of renal biopsy is remarkable to establish the diagnosis and rapidly initiate the treatment. Intensive immunosuppression is needed and, as our case shows, RTX may be an option, which allows control of the underlying disease and renal involvement.

## Authors’ contribution

MG and AM conducted manuscript redaction. DC; Patient management. FC; Histological diagnosis and manuscript revision. HS; Histological diagnosis. JS; Manuscript revision. FN; Manuscript revision.

## Conflicts of interest

The authors declare no conflict of interests.

## Funding/Support

None.
